# Supplementary material for: Determinants of podoconiosis among residents of Machakle District East Gojjam Zone Amhara Region Ethiopia
Source: PLoS Negl Trop Dis. 2023 Oct 5;17(10):e0011686. doi: 10.1371/journal.pntd.0011686 (PMC10581460; doi:10.1371/journal.pntd.0011686)
Supplement: Attachment — Submitted filename: PNTD-D-23-00471_reviewer.pdf [file pntd.0011686.s001.pdf]

PLOS Neglected Tropical Diseases

Determinantes of Podoconiosis among Residents in Machakle District East Gojjam Zone Amhara Region Ethiopia

--Manuscript Draft--

Manuscript Number:	PNTD-D-23-00471
Full Title:	Determinantes of Podoconiosis among Residents in Machakle District East Gojjam Zone Amhara Region Ethiopia
Short Title:	Determinantes of Podoconiosis
Article Type:	Research Article
Keywords:	Determinants; Machakel; Podoconiosis; Neglected Tropical diseases; Field Epidemiology; Ethiopia
Abstract:	Background<br><br>Podoconiosis (endemic non-filarial elephantiasis) is a chronic disease characterized by the development of persistent swelling of plantar foot initially; which progresses to the dorsal foot and lower leg slowly or in a number of acute episodes to reach the knee. About 4 million people are said to be affected by the disease worldwide and it is deemed a serious public health problem in at least 10 African countries including Ethiopia. Therefore this study aimed to identify the determinants of Podoconiosis among residence in Machakel district.<br><br>Method<br><br>Unmatched case control study design was conducted at Machakel district from August 30 to September 30/ 2022. The sample size was calculated using Epi-info software yielded 211 controls and 106 cases (317 study participants). Simple random sampling technique was used to select the cases using registration books of the district. Data were entered to Epi info version 7 and exported to SPSS version 22 for statistical analysis. Binary logistic regression was used to identify explanatory variables.<br><br>Result<br><br>A total of 312 study participants (104 cases and 208 controls) were included giving for a response rate of 98.42%. Bare foot (AOR, 5.83 [95% CI: 2.34-14.50]), female sex (AOR, 4.25 [95% CI: 2.22-8.14]), family history of podoconiosis (AOR 3.01(95% CI: 1.41-6.42) and age group 41-60 (AOR 5.05(95% CI: 2.35-10.83), and 61-80 AOR 15.74 95% CI: (5.56-44.55) were determinants of Podoconiosis.<br><br>Conclusion and recommendation<br><br>Barefoot, sex, family history of podoconiosis and age group were determinants of Podoconiosis. District health office should encourage for at risk populations especially for older age and family history of podoconiosis about shoe wearing practice in all their lives and do not exposed their skin and feet.
Additional Information:	
**Question**	**Response**
**Government Employee**<br><br>Are you or any of the contributing authors an employee of the United States government?<br><br>Manuscripts authored by one or more US	No - No authors are employees of the U.S. government.

Government employees are not copyrighted, but are licensed under a CC0 Public Domain Dedication, which allows unlimited distribution and reuse of the article for any lawful purpose. This is a legal requirement for US Government employees.<br><br>This will be typeset if the manuscript is accepted for publication.	
**Financial Disclosure**<br><br>Enter a financial disclosure statement that describes the sources of funding for the work included in this submission and the role the funder(s) played. This includes grants and any commercial funding of the work or authors.<br><br>This statement will be typeset if the manuscript is accepted for publication.<br><br>Review the submission guidelines and the instructions link below for detailed requirements and guidance.	No fund was received
**Competing Interests**<br><br>On behalf of all authors, disclose any competing interests that could be perceived to bias this work.<br><br>This statement will be typeset if the manuscript is accepted for publication.<br><br>Review the instructions link below and PLOS NTDs' competing interests policy to determine what information must be disclosed at submission.	The authors declare that they have no competing interests.
**Data Availability**<br><br>Provide a **Data Availability Statement** in the box below. This statement should detail where the data used in this	The datasets used and/or analysed during the current study are available from the corresponding author on reasonable request.

Powered by Editorial Manager® and ProduXion Manager® from Aries Systems Corporation

submission can be accessed. This statement will be typeset if the manuscript is accepted for publication.<br><br>Before publication, authors are required to make all data underlying their findings fully available, without restriction. Review our PLOS Data Policy page for detailed information on this policy. Instructions for writing your Data Availability statement can be accessed via the Instructions link below.	

Powered by Editorial Manager® and ProduXion Manager® from Aries Systems Corporation

Manuscript Click here to access/download;Manuscript;Manuscript.docx

Determinantes of Podoconiosis among Residents in Machakle District East Gojjam Zone Amhara Region Ethiopia

Teshome Tefera[1], Kassawmar Angaw Bogale[1], Yiteka Tegegn[1], Abebaw Gedef Azene[1], Kebadnew Mulatu[1], Gizachew Tadesse Wassie*[1]

[1]Department of Epidemiology and Biostatistics, School of Public Health, College of Medicine and Health Sciences, Bahir Dar University, Bahir Dar, Ethiopia

* Corresponding author

Po Box: 79

Abstract

Background: Podoconiosis (endemic non-filarial elephantiasis) is a chronic disease characterized by the development of persistent swelling of plantar foot initially; which progresses to the dorsal foot and lower leg slowly or in a number of acute episodes to reach the knee. About 4 million people are said to be affected by the disease worldwide and it is deemed a serious public health problem in at least 10 African countries including Ethiopia. Therefore this study aimed to identify the determinants of Podoconiosis among residence in Machakel district.

Method: Unmatched case control study design was conducted at Machakel district from August 30 to September 30/ 2022. The sample size was calculated using Epi-info software yielded 211 controls and 106 cases (317 study participants). Simple random sampling technique was used to select the cases using registration books of the district. Data were entered to Epi info version 7 and exported to SPSS version 22 for statistical analysis. Binary logistic regression was used to identify explanatory variables.

Result: A total of 312 study participants (104 cases and 208 controls) were included giving ~~for~~ a response rate of 98.42%. Bare foot (AOR, 5.83 [95% CI: 2.34-14.50]), female sex (AOR, 4.25 [95% CI: 2.22-8.14]), family history of podoconiosis (AOR 3.01(95% CI: 1.41-6.42) and age group 41-60 (AOR 5.05(95% CI: 2.35-10.83), and 61-80 AOR 15.74 95% CI: (5.56-44.55) were determinants of Podoconiosis.

Conclusion and recommendation: Barefoot, sex, family history of podoconiosis and age group were determinants of Podoconiosis. District health office should encourage ~~for~~ at risk populations especially ~~for~~ older age and family history of podoconiosis about shoe wearing practice in all their lives and ~~do~~ not exposed their skin and feet.

Key words: Determinants, Machakel**,** Podoconiosis, Neglected Tropical diseases, Field Epidemiology, Ethiopia.

Introduction

Podoconiosis is a chronic disease characterized by the development of persistent swelling of plantar foot initially; which progresses to the dorsal foot and lower leg slowly or in a number of acute episodes to reach the knee. Finally, the disease may end up in a permanent feature of elephantiasis of varying degree. The disease is common in families of barefooted agriculturalists of tropical Africa(1).

Podoconiosis (from the Greek word for foot: podos, and dust: konos) is unique in being an entirely preventable non-communicable tropical disease. In local communities, it is often called 'mossy foot disease', because the skin becomes rough and bumpy and its appearance resembles moss(2).

Podoconiosis has a curable pre-elephantiasis phase. However, once elephantiasis is established, podoconiosis persists and may cause lifelong disability(3). Podoconiosis (endemic non- filarial elephantiasis) has been recognized as a specific disease entity for over one thousand years and is widespread in tropical Africa, Central America and north India, yet it remains a neglected and under-researched condition(4). It is non-infective, usually crystalline blockage of the limb lymphatic, almost always affecting the lower limbs, especially the feet. It is widely distributed in a number of countries of tropical Africa, Central and South America, North India, Indonesia, Colombia, Ecuador, Brazil and Sri Lanka (5).

Podoconiosis (endemic non-filarial elephantiasis) is a geochemical disease occurring in individuals exposed to red clay soil of volcanic origin(6). The disease causes bilateral, but asymmetrical swelling almost invariably of the lower legs(7). Early symptoms of podoconiosis include itching of the skin of the forefoot and recurrent episodes of burning and oedema of the foot or lower leg, especially after periods of intense physical activity(8).

Although the aetiology is not fully understood, existing scientific evidence suggests the important role of exposure to irritant red clay soil in endemic areas as well as the effect of genetic susceptibility(9). Podoconiosis is found in highland areas of tropical Africa, Central

America and north-west India. Areas of high prevalence have been documented in Uganda (10), Tanzania (11), Kenya (12), Rwanda, Burundi, Sudan and Ethiopia (13), and in Equatorial Guinea (14), Cameroon (15), the islands of Bioko, Sao Tome & Principe (16) and the Cape Verde islands. And it is related to poverty. Studies have also indicated that podoconiosis exists in areas where the altitude is above 1000meters above sea level and annual rainfall above 1000 millimetres. About 4 million people are said to be affected by the disease worldwide and it is deemed a serious public health problem in at least 10 African countries(1,17).

Podoconiosis is a multifactorial disease with evidence of genetic susceptibility and environmental exposure(18). Although the ethology is not fully understood, current evidence suggests genetic susceptibility and the role of mineral particles from irritant volcanic soils supposed to cause podoconiosis(19).The disease only affects some barefoot individuals; not all exposed individuals develop podoconiosis.

Podoconiosis follows a chronic course causing progressively increasing disability with continued exposure to irritant soils. It results in bilateral progressive chronic swelling of the lower legs, usually limited below the level of the knees. The pathogenesis of the disease has not yet been investigated in depth, but it is believed to be caused by fine particles in the soil that penetrate the skin and induce an inflammatory reaction in the lymphatic system(20). However, early stage disease can easily be treated by foot hygiene, bandaging and shoes.

Podoconiosis is classified into five stages where the first stage swelling is limited to below the ankle and is reversible overnight. The second stage swelling is not reversible, and when bumps and knobs are present they remain below the level of the ankle. In the third stage of the disease, bumps and knobs are found above the level of the ankle. The fourth stage entails above knee swelling whereas the fifth stage involves joint fixation as a result of surrounding soft tissue overgrowth(20).

Podoconiosis has recently been included in the World Health Organization's Neglected Tropical Diseases (NTDs) list(21). Areas of high prevalence of podoconiosis have been documented in tropical Africa, Central America and north India(22). Of affected countries, Ethiopia appears to have the highest number of people with podoconiosis(23), with 11 million people at risk through exposure to irritant soil, and an estimated 1 million people affected countrywide(24). In Ethiopia, prevalence estimates range from 2.8 to 7.4% in endemic areas(24-26). Podoconiosis can be prevented, early forms of the disease can be

treated, and disease progression can be controlled with simple but effective measures such as washing feet with soap and water on a regular basis and wearing protective shoes consistently(1). Hence this study aimed to study the determinants of podoconiosis in Machakel district Ethiopia.

Methods and Materials

Study Area

This study was conducted in Machakel district. Machakel district is one of the districts in East Gojjam zone, Amhara national Regional State. It is found at a distance of 328 km North West from Addis Abeba, the capital city of Ethiopia, 237 km far from Bahir Dar, the capital city of Amhara Region. Machakel woreda is bordered ~~by~~ on the North by Bibugn district, on the South by Debre Elias district, on the Northwest by Sinan, on the Southwest by Gozamen and on the East by Dembecha (West Gojjam).According to the 2014 E.C projected census 2007, the total population is 146,942 from which about 73,618(50.1%) are female and 73,324(49.9%) male population. Machakel district has 30 kebeles. In the Machakel district, there are 24 health posts and six health centres all are providing health service.

Study design and period

Unmatched case control study design was conducted from August 30 to September 30 ~~/~~ 2022.

Source and Study population

All resident of Machakel district were the source population. Whereas the study population was all adult patients age 18 and older ~~those who~~ identified and registered as podoconiosis case by the district and ~~those~~ neighbouring individuals without podoconiosis.

Cases: Were person of age 18 and old, resided in ~~all~~ kebeles of Machakel district and who had diagnosed and registered as podoconiosis case by the district.

Controls: Were person ~~with~~ age 18 years and old who ~~had~~ not podoconiosis after ~~diagnosed~~ by clinical nurses ~~clinically~~ and who lives in the neighbouring house to podoconiosis patients (case).

Inclusion criteria

Cases: were residents of Machakel district and those registered as podoconiosis cases of age 18 years and old resident in the study area for at least for the last six months were included.

Controls: were those age of 18 years and old, resident of Machakel district, who had not developed podoconiosis diseases and neighbour to a podoconiosis patient (case).

Exclusion criteria

Cases: Severely ill and bed riddle cases, age less than 18years and resided less than six months was excluded during the data collection period.

Controls: Those people without podoconiosis and not being neighbour to the cases were excluded.

Sample size determination

The sample size was calculated using Epi-info 7 software based on the assumption of 95% confidence interval, 85% power, control to case ratio of 2:1, ~~present~~ of exposed family history among controls 11.4%, odds ratio to be detected as 2.81(15) and non-response rate of 10% yielding 211 controls and 106 cases (317 study participants).

Sampling Techniques

Simple random sampling technique was used to select the cases from podoconiosis registration books of Machakel woreda health office as a sampling frame. The control groups were included from the community and Simple random sampling technique was used to

select the control. Those controls were selected from neighbouring of the cases by lottery method.

Variables

Dependent variable

- ✓ Podoconiosis case(yes/no)

Independent variables

- ✓ **Socio-demographic Characteristics** such as: Age, Sex, Residence, Income, Educational status, Occupation,
- ✓ **Behavioural related factors :** Feet washing practice, and Shoe Wearing practice
- ✓ **Family history:**1rst degree, 2nd degree, 3rd degree and others family

Operational Definition

Cases: Those podoconiosis cases registered by the district as a podoconiosis case and found on the sampling frame.

Controls: A person who had not podoconiosis disease after diagnosed by the clinical nurses clinically and neighbours of cases.

Feet washing: patient daily wash their foot with soap.

Barefoot: not wearing any type of shoe every day.

Family history: History of podoconiosis in the family clustering, such as,1st degree(parents, child), 2nd degree(grandparents, siblings), 3rd degree(aunt, uncle, nephew, cousin, niece), other (husband and wife)(11).

Shoe wearing: wearing a full covering shoe every day during each activity.

Data collection tools and techniques

Data were collected using interviewer administered questionnaire it was prepared in English and translated into Amharic language for the purpose of community level and back to English for checking language consistency. Data was collected by clinical nurse. The whole data collection process was supervised by health officers.

Data quality control

Questionnaire were prepared in English version by reviewing different literatures and translated in to Amharic (local language) and back to English by different language experts for consistency. Steps were taken to ensure the quality of this work. Pre-test was conducted on 16(5%) study participants in another district and necessary corrections were made on the questionnaire. The supervisors and principal investigator closely following the day to day data collection process to ensure completeness and consistency of the collected questionnaires on a daily basis. Training was given for the data collectors and supervisors, and the whole data collection process was closely supervised.

Data processing and analysis

Prior to analysis, the whole data was cleaned and checked for completeness. Errors related to inconsistency was verified using cross tabulation and other data exploration methods .After the completeness of the data checked; it was entered into Epi-Info version 7.2.1.0 software packages and transferred to SPSS version 22 software for analysis. Descriptive statistics was used to give a clear picture of background variables like age, sex, and other variables. The frequency distribution of both dependent and independent variables were done. Binary logistic regression was used to identify the determinants of Podoconiosis and the single independent variable with the outcome variable. Hosmer-Lemeshow test was used to check goodness of fit of the model. Variables having an association with the outcome variable a p-value of less than 0.2 were considered in the multivariable logistic regression analysis.

Adjusted odds ratios (AORs) with 95% confidence intervals were used to show association between explanatory variables and a dependent variable. Those independent variables with P-value < 0.05 was considered statistically significant factors associated with outcome variable.

Results

Socio-demographic characteristics of participants

A total of 312 participants (104 cases and 208 controls) were included, ~~giving for~~ a response rate of 98.42%. The mean age of respondents was 47.21 years (SD 11.47 years). Nearly two third 229(73.32%) were farmers by occupation. The mean age of the cases was 53.8 years (SD=15.43 years). Female constitute 67.30 % of cases, 58(55.76 %) of cases had family history of Podoconiosis, the proportion of bare foot among cases was 30.76% and 76.92% of cases ~~were can not~~ read and write.

The mean age of controls was 43.88 years (SD 9.5 years). Having family history of Podoconiosis was recorded in (57.69%), bare foot (11.53%), ~~Female~~ (44.71%) and ~~of~~ farmer ~~occupation~~ constitutes (71.15 %) of controls (Table 1).

Table 1: Socio-demographic characteristics of study participants in Machakel district North-West Ethiopia, 2022.

~~Characteristics~~	Category	Cases (n (%))	Controls (n (%))
**Sex**	Male	34(54.8)	115(60)
	Female	70(45.19)	93(44.71)
**Age**	21-40	14(13.46)	93(44.71)
	41-60	63(60.57)	101(48.55)
	61-80	27(25.96)	14(6.73)
**Residence**	Urban	16(15.38)	29(13.9)
	Rural	88(84.6)	179(86.05)
**Marital status**	Unmarried+	32(30.7)	13(6.25)

	Married	72(69.23)	195(93.75)
**Occupation**	Merchant	4(3.8)	11(5.25)
	Farmer	81(77.88)	148(71.15)
	House wife	16(15.38)	47(22.59)
**Education**	Not read and wright	80(76.92)	112(53.84)
	Read and wright	21(20.19)	67(32.21)
	~~primary~~	2(1.92)	28(13.65)
	Higher	1(0.96)	0(0)
**Income**	500-1500	48(46.15)	48(23.41)
	1501-3500	43(41.34)	118(57.56)
	>3500	13(12.5)	42(20.2)

Behavioural related factors of participants

The proportion of bare foot among cases was 32(30.76%) whereas it was 24(11.53%) among controls. About 84(80.76%) of cases and 188(90.38%) of controls did wash their feet 6-8 times per week and 52(50%) of cases, 152(73.07%) of controls wash their feet 6-8 times per week with soap.

Among cases 26(25%) and 9(8.6%) did wear shoe at work and at home respectively. Whereas 88(42.30%) and 38(18.26%) ~~were~~ among controls, ~~respectively.~~ Regarding mechanism of feet washing, among cases 84(80.76%) washed their legs with water only. About 188(90.38%) controls washed their legs with water only. Among cases 12(11.5%) and controls 120(57.69%) start shoe wearing between 1-20 years of age. About 58(55.76%) of cases and 120(57.69%) controls had family history of podoconiosis (Table 2).

Table 2: Behavioural related factors of podoconiosis in Machakel district North-West Ethiopia, 2022

Characteristics	Category	Cases (n (%))	Controls (n (%))
**Having enough water for washing**	Yes	104(100)	208(100)
**Washing feet**	1-2 times	2(1.92)	0(0)

**per week**	3-5 times	17(16.34)	13(6.25)
	6-8 times	84(80.76)	188(90.38)
	>8	1(0.96)	7(3.36)
**Washing feet per week with soap**	1-2 times	5(4.80)	10(4.80)
	3-5 times	33(31.73)	35(16.82)
	6-8 times	52(50)	152(73.07)
	>8	0(0)	2(0.96)
**Barefooted**	Yes	32(30.76)	24(11.53)
	No	72(69.23)	184(88.46)
**Starting year of shoe wearing**	1-20 year	12(11.53)	120(57.69)
	21-40 year	53(50.96)	59(28.36)
	41-60	7(6.73)	5(2.40)
**Years with shoe wearing**	All years	30(28.84)	181(87.01)
	More than have the year	22(21.15)	2(0.96)
	Half year	16(15.38)	1(0.48)
	Less than year	4(3.84)	0()
**Days with shoe from the week**	Every day	47(45.19)	194(93.26)
	More than 5 day	22(21.15)	4(1.95)
	2-5 day	35(33.65)	10(4.80)

Factors Associated with Podoconiosis

Bare foot, sex, family history of podoconiosis, occupation, marital status, Age and owned a pair of shoes were found to be associated with Podoconiosis in the uni-variable binary logistic regression analysis at p values< 0.2.

From multivariable logistic regression analysis, Sex, bare foot, family history of podoconiosis and age were found to be associated with Podoconiosis with 95% CI, at p-value <0.05 statistical significance level. The goodness-of-fit statistics for the model were assessed by using the Hosmer-Lemeshow test, with a p-value of 0.97.

The odds of having Podoconiosis was 5.83 times higher in bare footed persons (AOR 5.83, 95% CI: 2.34-14.5) as compared who wore shoes. The odds of having Podoconiosis 4.25 times higher in females (AOR 4.25: 95% CI: 2.22-8.14) as compared to males. The odds of having Podoconiosis was 3.01 times higher among participants who had family history of podoconiosis [AOR= 3.01, 95% CI: 1.41-6.42] as compared to that of those who had no family history of podoconiosis. The odds of having podoconiosis was 2.05 times higher in age group 41-60(AOR=5.05, 95% CI: 2.35-10.83) and 15.74 times higher in age group 61-80 (AOR=15.74, 95% CI: 5.56-44.55) as compared to age less than 40 (Table 3).

Table 3: Factors associated with podoconiosis in Machakel district North-West Ethiopia, 2022.

**Variables**	**Category**	**case**	**Control**	**COR [95% CI]**	**AOR [95% CI]**
Bare foot	No	72	184	1	1
	Yes	32	24	3.40(1.87-6.17)	5.83(2.34-14.50)*
Sex	Female	70	93	2.54(1.55-4.16)	4.25(2.22-8.14)*
	Male	34	115	1	1
**Marital status**	Single	3	6	1	1
	Married	80	195	0.82(0.2-3.36)	0.19(0.03-1.11)
	Divorced	11	3	7.33(1.11-48.26)	0.83(0.09-7.5)
	Widowed	10	4	5.00(0.82-30.46)	0.34(0.04-2.94
Age group	21-40	14	93	1	1
	41-60	63	101	4.14(2.17-7.89)	5.05(2.35-10.83)*
	61-80	27	14	12.81(5.44-30.14)	15.74(5.56-44.55)*
Family history	No	46	88	1	1
	Yes	58	120	1.09(1.21-3.11)	3.01(1.41-6.42)*
Occupation	Farmer	81	148	0.36(0.06-2.22)	0.62(0.07-5.35)
	House wife	16	47	0.22(0.03-1.48)	0.27(0.02-2.74)
	Merchant	7	13	1	1
Owned a pair of shoe	No	4	3	2.73(0.6-12.44)	2.5(0.38-16.53)
	Yes	100	205	1	1

1=Reference, *=significant at p-value <0.05 in multivariable logistic regression

Discussion

Even though podoconiosis has been known for more than a millennium, it has been neglected and under-researched. Podoconiosis has recently been designated neglected tropical disease status by the WHO(25) .

Bare footed people were at higher risk to develop podoconiosis as compared to that of their counterparts. This implies that bare footed individuals might be exposed to irritant minerals found in earth. This leads to Podoconiosis. This finding was agreed with the study conducted in Southern Ethiopia(26). This might be due to the reason that barefooted individuals may allow mineral particles from the soil to enter in to their feet that can possibly initiate the pathology of the disease(27, 28).

Being female was another variable with higher odds among cases compared to Males. This finding agreed with a study done in West and East Gojjam zone shows that; The odds of Podoconiosis was lower risk in males (15). This is might be due to variation in exposure in males and females in these areas. Traditionally males usually wear shoes frequently than females.

Family history of podoconiosis was also at higher odds among cases than controls. This might be due to the effect of genes in the development of Podoconiosis(29). This study was in line with a study done in West and East Gojjam zone (35).

Older age, particularly people aged 41-60 years and 61-80 years were at higher risks to develop podoconiosis as compared to those with age group less than 40 years. This implies that older age individuals might have long-term exposure to irritant minerals on the earth that may increase the risk of developing podoconiosis.

Limitation of the study

The limitation of this study was the possibility of recall bias that might have been introduced due to retrospective nature of the study design.

Conclusion

Podoconiosis is a common but neglected tropical disease leading to dramatic non filarial elephantiasis in the tropics region. This study showed that the determinant factors of Podoconiosis are regular walking on barefoot, family history of podoconiosis, female sex and older age. People should be encouraged to wearing shoes all the time. Particularly, females in the study area and other similar settings should be given attention in the ~~existed~~ primary health care packages to promote shoes wearing as males.

Declarations

Ethics approval and consent to participate

The ethical clearance issues of this study were reviewed and approved by the Ethical Review Committee (IRB) of the College of Medicine and Health Sciences, Bahir Dar University. Permission letter was also obtained from Amhara Public Health Institute (APHI) before the actual data collection; permission was taken from East Gojjam zone health department and Machakel woreda health office. Written informed consent was taken for each participant. Confidentiality was kept and their name was changed to codes.

Consent for publication

Not applicable

Availability of data and material

The datasets used and/or analysed during the current study are available from the corresponding author on reasonable request.

Competing interests

The authors declare that they have no competing interests.

Funding

No fund was received

Authors' contributions

TT conceived and designed the research protocol. KA, YT, AG, KM and GT approved the proposal with extensive revisions, participated in the data analysis, and had written the manuscript. All authors have read and approved the final manuscript.

Acknowledgment

We would like to thank Machakel District Health Office workers for giving me baseline and supportive letter for data collection. Also, we would thank the data collectors and the study participants.

References

1. Tekola F MD, Davey G. Economic costs of endemic non-filarial elephantiasis in Wolaita Zone, Ethiopia. Trop Med Int Health. 2006.
2. Yakob B, K. Deribe, and G. Davey,. Health professionals’ attitudes and misconceptions regarding podoconiosis: potential impact on integration of care in southern Ethiopia. Transactions of the Royal Society of Tropical Medicine and Hygiene,. 2010.
3. Tekola F, et al., . Development and testing of a de novo clinical staging system for podoconiosis (endemic non-filarial elephantiasis),. Tropical Medicine & International Health,. 2008.
4. Geshere G TF, Petros B. Parasitological,. serological and clinical evidence for high prevalence of podoconiosis (non-filarial elephantiasis) in Midakegn district, central Ethiopia. Tropical Medicine and International Health,. 2012; .
5. Nenoff P CJ, Muylowa K, Davey G. . Podoconiosis-non-filarial geochemical elephantiasis-a neglected tropical disease. journal of German society of dermatology ,. 2010; .
6. Desta A CM, Hendric D, Emi Watanabe,Tsega G et al., .

A qualitative study exploring barrier related to use of foot wear in rural highland Ethiopia,. 2013.
7. Wanji S TN, Esum M, Che JN, Mand S, et al. , .

demic elephantiasis of the lower legs in tropical Africa,. 2008.
8. Elephantiasis (Elephantitis) Diseases, Types, Pictures, Treatment,.
9. Kebede D ST, Fasil TokolaAyele, Richard O.Phillips ,, .

ten years of Podoconiosisreaserch in Ethiopia,. 2013.
10. E.W. P. The association of endemic elephantiasis of the lower legs in east Africa with soil derived from volcanic rocks,. 1976; .
11. WHO. Diseases covered by NTD Department, Book Diseases covered by NTD Department,. 2011.

12. Price E. Podoconiosis. Non-filarial Elephantiasis,. Oxford Medical Publications. 1990.
13. Cohen LB. idiopathic lymphoedema of Ethiopia and Kenya,. East Africa Medical Journal. 1960.
14. Academic Medical Centre / University of Amsterdam A, the Netherlands, *corresponding author. Podoconiosis, a neglected tropical disease. 2012.
15. Elfu B. Determinants of Podoconiosis, . 2017
16. Yordanos B ST, Tsige A, Abreham T, Gail D. Patients' perceptions of podoconiosis causes, prevention and consequences in East and West Gojam, Northern Ethiopia. . 2011; .
17. EW. P. Endemic elephantiasis of the lower legs in Rwanda and Burundi,. 1976.
18. Ruiz L, E Campo, M Corachan: . Elephantiasis in Sao Tome and Principe,. 1994.
19. ARGACHEW Y. ASSOCIATION BETWEEN FOOT TYPE AND PODOCONIOSIS,. 2018.
20. Mousley E, et al., . The impact of podoconiosis on quality of life in Northern Ethiopia. Health and quality of life outcomes,. 2013.
21. Sime H DK, Assefa A, Newport MJ, Enquselassie F, Gebretsadik A, et al. Integrated mapping of lymphatic filariasis and podoconiosis: lessons learnt from Ethiopia,. 2014.
22. Frommel D AB, Pfeifer HR, Sanchez A, Frommel A, Mengistu G. . Podoconiosis in the Ethiopian Rift Valley,. 1993.
23. Feven Dejene1 HM, Henok Asefa2. podoconiosis and associated factors in Dano district, . 2019.
24. Feven Dejene1 HM, Henok Asefa2. associated factors of podoconiosis,. 2019.
25. Organisation WH. Podoconiosis, a neglected tropical disease. 2011.
26. Meskele AEHYM KE. Podoconiosis Prevalence and Its Associated Factors in Soddo Zuria District,.
27. Minstory of Health(MoH). Lymphatic filariasis and podoconiosis morbidity management and disability prevention guidelines,. 2016.
28. Muli J GJ, Kagai J. Soil iron and aluminium concentrations and feet hygiene as possible predictors of Podoconiosis occurrence in Kenya,. 2017.
29. (Nenoff P CJ, Muylowa K, Davey G. Podoconiosis-non-filarial geochemical elephantiasis-a neglected tropical disease,. 2010.
