# Supplementary material for: Determinants of podoconiosis among residents of Machakle District East Gojjam Zone Amhara Region Ethiopia
Source: PLoS Negl Trop Dis. 2023 Oct 5;17(10):e0011686. doi: 10.1371/journal.pntd.0011686 (PMC10581460; doi:10.1371/journal.pntd.0011686)
Supplement: Attachment — Submitted filename: Point by point response letter to Reviewers.docx [file pntd.0011686.s003.docx]

**Point by Response to the Reviewers**

Dear editor, thank you for considering our manuscript for publication; we have revised the contents based on the reviewer’s comments as below.

Ref: Submission ID: (PNTD-D-23-00471) - [EMID:d20f5e4903eb5d45]

Title: "Determinants of Podoconiosis among Residents in Machakle District East Gojjam Zone Amhara Region Ethiopia" for consideration at PLOS Neglected Tropical Diseases.

Reviewer's Responses to Questions

-Are the objectives of the study clearly articulated with a clear testable hypothesis stated?</br>

-Is the study design appropriate to address the stated objectives?</br>

-Is the population clearly described and appropriate for the hypothesis being tested?</br>

-Is the sample size sufficient to ensure adequate power to address the hypothesis being tested?</br>

-Were correct statistical analysis used to support conclusions?</br>

-Are there concerns about ethical or regulatory requirements being met?</br></br>

Reviewer #1: • The authors stated the objective of the study. However, the writers have not spelt out the justification of the study. I suggest the writers give a solid and convincing reasons for this study of which findings has already been established in literature.

Response:

•	Authors have obtained permission from the appropriate ethic regulatory bodies for the study. Please let the ‘ethics approval and consent to participate’ statement appear at the methods section of the manuscript and not to the end. Also, kindly provide the ethical clearance reference number of the study in the ethic declaration statement.

*Response: Thank you for your feedback. We presented ‘ethics approval and consent to participate in the method section. Hence, the ethical clearance reference number of the study was* 523/2022*(Page No. 9, line #199-206).*

•	The authors clearly described the appropriate population suitable for the study. However, the description of cases and control, inclusion and exclusion criteria are not clear and should be stated with clarity.

*Response: We have revised the description of cases and controls (page 5,6, Line #121-126)*

•	The variable; ‘Family history’. Line 153 is not clear. Please kindly clarify this.

Response: It was intended to say whether participants had family history of podoconiosis or not from their 1^st^, 2^nd^, 3^rd^ or other families. We have revised it in the manuscript document too (page 7,line #158-160).

•	The sample size was determined from sample size calculation and is sufficient to ensure adequate power to address the hypothesis being tested.

*Response: Thank you for your acknowledgements.*

Reviewer #2: The methodology described in the manuscript is appropriate for the clearly stated objective. Ethical requirements were fulfilled for for the study.

--------------------

<b>Results</b></br></br>

-Does the analysis presented match the analysis plan?</br>

-Are the results clearly and completely presented?</br>

-Are the figures (Tables, Images) of sufficient quality for clarity?</br></br>

Reviewer #1: • The results are not completely presented. I kindly suggest the following:

-	Table headings should be bolded

-	Table 1 and 2 are overlapping. I suggest that if a table cannot fit into one page, the authors should break that into sections. Example; Table 1, Table 1 (continued) maintaining the title of the columns under each table sections.

*Response: Thank you for the reminder. We bolded table headings as your recommendations. But table 1 and Table 2 are a bit different. In table 1 the socio-demographic characteristics of participants were presented. Whereas, in Table 2 behavioral factors were presented.*

-	Table 1: The categories under ‘education is not clear’. I suggest the writers put the categories as: Cannot read and write, Can read and write. Primary, Higher.

*Response: We have revised as your suggestions (Table 1).*

-	Please in which currency is the characteristic ‘Income’ categorized? I suggest the authors provide this information in parenthesis beside ‘Income’. Example: Income (USD)

*Response: We have revised that the currency is Ethiopian Birr.*

-	Table 2: Please the statement in Line 213-214 cannot be found in the referenced table (Table 2)

Response: *Thank you for your reminder. We have included in table 2.*

-	Please what is the difference between the result mentioned in Line 210-211 and Line 214-216? Please clarify.

*Response: Thank you for your comment. It was duplication. So we have deleted lines 214-216.*

-	The characteristic ‘Washing feet per week’, is it without soap? If so, please correct it.

Response: *Yes, it washing feet per week without soap (table 1)*

-	I think the characteristic ‘Years with shoe wearing should be ‘Number of years of shoe wearing’

*Response: Revised as your suggestion*

-	Please the categories under the characteristic ‘Year with shoe wearing’ is not clear. The arrangement of categories is not chronological. What is the difference between ' More than half year and Less than a year? Please correct this statement and let the categories be chronologically presented.

*Response: We have re-categorized in to three categories by merging the last two categories.*

-	The characteristic ‘Days with shoe from the week’ not clear. I suggest it should be presented as ‘Number of days of shoe wearing per week’

*Response: We have revised as your suggestions.*

-	Line 235-236: The statistic; 2.05 mentioned in the statement ‘The odds of having podoconiosis was 2.05 times higher….’is not found in the referenced Table 3. Please correct this.

*Response: Thank you. It was editorial error. We corrected as 5.05 as shown in table 3.*

-	Table 3: Please provide a column containing information on the P- values

*Response: Thank you for your comments. But we preferred to present as it stands now to prevent table congestion. Besides, 95% CI could give the information P-values could give.*

Reviewer #2: The results are clearly presented.

*Response: Thank you for your acknowledgements.*

--------------------

<b>Conclusions</b></br></br>

-Are the conclusions supported by the data presented?</br>

-Are the limitations of analysis clearly described?</br>

-Do the authors discuss how these data can be helpful to advance our understanding of the topic under study?</br>

-Is public health relevance addressed?</br></br>

Reviewer #1: • The authors provided conclusions which are supported by the data presented in the manuscript.

•	The limitation of the study is mentioned.

•	The benefit of the findings to the advancement of knowledge on the topic under study have been discussed and public health relevance has also been touched on.

*Response: Thank you for your acknowledgements.*

Reviewer #2: The conclusions are supported by the data presented and study limitation was also noted.

--------------------

<b>Editorial and Data Presentation Modifications?</b></br><br/>

Use this section for editorial suggestions as well as relatively minor modifications of existing data that would enhance clarity. If the only modifications needed are minor and/or editorial, you may wish to recommend “Minor Revision” or “Accept”.

Reviewer #1: I suggest the authors take into consideration the recommendations made in the results section to make them look more presentable.

*Response: We acknowledge the reviewer’s recommendations so that we have addressed all comments.*

Reviewer #2: (No Response)

--------------------

<b>Summary and General Comments</b></br></br>

Use this section to provide overall comments, discuss strengths/weaknesses of the study, novelty, significance, general execution and scholarship. You may also include additional comments for the author, including concerns about dual publication, research ethics, or publication ethics. If requesting major revision, please articulate the new experiments that are needed.

Reviewer #1:

• At present, the manuscript needs some further work:

1. The manuscript is poorly written with typographical and grammatical errors making it difficult to read and understand. I therefore highly recommend language editing

*Response: Thank you for your constructive feedbacks. We have revised the entire manuscript for grammar and typo errors.*

2. I presume the word ‘Determinantes’ in the title is supposed to be an English word ‘Determinants’. Please correct.

*Response: Corrected*

3. The font type and size of the main write up is different from the one used for the reference lists. I suggest authors edit the manuscript to have a uniform font type and size.

*Response: we have edited for the font type and size.*

4. The ‘Author’s summary’ as required by the journal is missing in the manuscript. I suggest the authors provide this important information after the ‘Abstract’ statement

*Response: We added in the right place (page 3, line numbers 40-48).*

5. The in-text citation is incorrect. The journal requires a square bracket not parenthesis

*Response: Thank you for your suggestions. We corrected as your suggestions.*

6. Most of the bibliographies are incorrect. For example, some are incomplete, some in capital letters etc. I recommend that the authors edit the bibliographies to suite the journal’s style.

*Response: We corrected the whole bibliographies.*

Reviewer #2: The study provides important information on determinant of a neglected NTD and adds to the limited body of knowledge on this disease.

However, this reviewer will recommend that authors should consider employing the service of an English language editor. The grammar usage in the manuscript is currently not at an acceptable level for publication.

*Response: Thank you for your constructive feedbacks. We have revised the entire manuscript for grammar and typo errors.*

-------------------------------------------------------//-------------------------------------------
